# Gender Disparities in Ischemic Heart Disease Management: Underdiagnosis in Women and Differences in Treatment

**DOI:** 10.7759/cureus.89912

**Published:** 2025-08-12

**Authors:** Younes El Bassiri, Amta Azeem, Aviral C Sharma, Mera Hassan, Mariam Hassan, Ibrahim Omari

**Affiliations:** 1 School of Medicine, Wenzhou Medical University, Wenzhou, CHN; 2 School of Medicine, Ponce Health Sciences University, St. Louis, USA; 3 Internal Medicine, Saba University School of Medicine, The Bottom, BES; 4 General Surgery, Sheba Medical Center, Ramat Gan, ISR

**Keywords:** cardiac rehabilitation, coronary microvascular dysfunction, healthcare disparities, hydroxymethylglutaryl-coa reductase inhibitors, ischemic heart disease, nonobstructive myocardial infarction, percutaneous coronary intervention, sex factors, spontaneous coronary artery dissection, women’s health

## Abstract

Ischemic heart disease (IHD) remains the leading cause of death globally, with mounting evidence of gender disparities in its diagnosis, management, and outcomes. Women are frequently underdiagnosed and undertreated despite having similar or greater cardiovascular risk compared to men. These disparities contribute to delayed care, suboptimal treatment, and poorer short- and long-term outcomes in women.

A narrative review was conducted using peer-reviewed literature published between 2015 and 2025. The search focused on gender differences in IHD presentation, diagnosis, pharmacologic and interventional treatment, and specific conditions such as myocardial infarction with non-obstructive coronary arteries (MINOCA) and spontaneous coronary artery dissection (SCAD). Articles providing quantitative data, clinical observations, or expert consensus on gender disparities in IHD were prioritized.

Our results indicate that women are less likely to receive diagnostic imaging, percutaneous coronary intervention, and statin therapy, even when presenting with comparable clinical indicators. Atypical symptom presentations and a higher prevalence of non-obstructive disease, including MINOCA and SCAD, contribute to underdiagnosis. Women with ST-elevation myocardial infarction also have higher rates of bleeding complications and 30-day mortality, emphasizing the need for tailored therapeutic protocols. Cardiac rehabilitation participation is significantly lower among women due to systemic barriers such as referral bias, socioeconomic factors, and program design not tailored to women’s needs. Clinical trials continue to underrepresent women, limiting sex-specific evidence to guide care.

Persistent gender disparities in IHD highlight the urgent need for sex-specific diagnostic pathways, treatment protocols, and greater representation of women in cardiovascular research. Enhancing clinician awareness, modifying existing guidelines, and restructuring clinical trials to include sex-disaggregated analysis are essential. These efforts are critical to improving cardiovascular outcomes and achieving equity in IHD care for women.

## Introduction and background

Background

Ischemic heart disease (IHD) is the foremost cause of death worldwide, affecting men and women differently. Despite comparable or higher risk profiles, women are underdiagnosed and undertreated in clinical practice, leading to poorer outcomes [1,2]. Studies consistently show that women are less likely to be referred for diagnostic testing or receive timely interventions, even when presenting with significant risk factors [2]. This underrecognition contributes to delays in care and higher rates of complications following cardiac events [1]. Addressing this gap is essential for reducing the burden of cardiovascular disease in women and improving survival outcomes. 

The disparity in care is driven in part by gender differences in disease presentation, diagnostic evaluation, and therapeutic management [3,4]. Women frequently present with non-classical symptoms, complicating the diagnostic process and increasing the likelihood of misdiagnosis [3]. In treatment, they are less likely to undergo percutaneous coronary intervention (PCI) or to be prescribed statins at guideline-recommended doses [4]. Additionally, conditions like myocardial infarction with non-obstructive coronary arteries (MINOCA) and spontaneous coronary artery dissection (SCAD) are more prevalent in women and often overlooked during standard evaluations [5,6]. These clinical entities require a higher degree of suspicion and specialized management approaches to ensure appropriate care [5,6].

This review synthesizes findings from observational studies, randomized controlled trials, national registry data, and expert consensus statements published between 2015 and 2025, prioritizing peer-reviewed literature reporting sex-stratified outcomes. This review aims to analyze gender disparities in IHD diagnosis and treatment, focusing on underdiagnosis in women, differences in intervention and pharmacotherapy, and their impact on cardiovascular outcomes. By drawing from recent literature, this discussion will highlight patterns of clinical inequity and explore the implications of these disparities on long-term health. The review will also examine how existing clinical pathways contribute to differential treatment, particularly in the use of PCI and statin therapy. While gender is the primary focus, disparities in IHD care are often compounded by intersecting factors such as race, socioeconomic status, and age. Structural contributors such as provider bias, limited access to specialized care, and insurance-related barriers further amplify these inequities. Ultimately, this review seeks to inform more equitable practices in cardiovascular care and advocate for greater integration of sex-specific approaches in both diagnosis and treatment.

## Review

Methods

A comprehensive literature search was performed across the past 10 years (2015-2025) to gather data related to gender disparities in IHD, including underdiagnosis, treatment differences, and outcomes. Emphasis was placed on peer-reviewed articles providing direct evidence or systematic reviews. Quotations and data from these articles were extracted to support findings.

This was a narrative (non-systematic) review. Literature searches were conducted using three major databases: PubMed/MEDLINE, Scopus, and Web of Science. Additional sources were identified through hand-searching of reference lists from relevant guidelines and review articles.

Search terms included combinations of: (“ischemic heart disease” OR “coronary artery disease” OR “acute coronary syndrome”) AND (“sex differences” OR “gender disparities” OR “women” OR “female”) AND (“diagnosis” OR “treatment” OR “outcomes” OR “statins” OR “PCI” OR “cardiac rehabilitation” OR “MINOCA” OR “SCAD”). Boolean operators such as AND and OR were used to broaden and refine search results.

Inclusion criteria were as follows: peer-reviewed articles published between 2015 and 2025; English language; human subjects; and studies reporting sex- or gender-specific data related to IHD diagnosis, treatment, or outcomes. We included observational studies, randomized controlled trials, systematic reviews, and expert consensus statements. Exclusion criteria included non-English articles, pediatric populations, non-cardiac studies, and commentaries or editorials unless published by a professional society.

Title and abstract screening was conducted by the primary author, and full texts were reviewed where necessary. All co-authors reviewed the final selection of sources. Although no formal quality appraisal tool was applied, emphasis was placed on high-impact journals, large-scale studies, and recent publications to reduce selection bias.

Key findings and quotations were extracted and synthesized thematically by topic, including diagnostic disparities, interventional gaps, pharmacologic treatment, cardiac rehabilitation, and clinical trial participation.

Results

Underdiagnosis of IHD in Women

Women with IHD often present with atypical symptoms and are more likely to have ischemia without obstructive coronary artery disease (INOCA), complicating diagnosis. Studies estimate that INOCA affects up to 65% of women evaluated for chest pain but found to have non-obstructive coronary arteries, compared to approximately 30% in men [7]. Advanced imaging has demonstrated that women frequently present with ischemia in the absence of obstructive coronary artery disease, necessitating tailored diagnostic pathways [7]. The lack of tailored diagnostic algorithms places women at risk of underdiagnosis [8].

Women with non-obstructive coronary artery disease (CAD) often face underdiagnosis because current diagnostic algorithms are not specifically designed for identifying ischemia with INOCA in women [8]. Moreover, utilizing cardiac imaging techniques that account for sex differences can enhance both the accuracy of diagnosis and the management of IHD in women [9].

Further discussion on advanced imaging, including cardiac MRI (CMR), PET, and coronary CTA, and their utility in detecting perfusion abnormalities and coronary microvascular dysfunction, is provided in later sections [10].

Recent estimates from the Global Burden of Disease (GBD) 2021 highlight these disparities on a population level, showing that although women experience fewer IHD-related deaths than men, they carry a disproportionately higher burden of disability, as summarized in Table 1.

**Table 1:** Global Sex-Disaggregated Burden of Ischemic Heart Disease (GBD 2021)

Metric	Women	Men	Source
Deaths (2021)	~4.94 million (part of 8.99 million total deaths)	~5.68 million	[11,12]
Disability-Adjusted Life Years (DALYs)	Slightly lower overall than men	Slightly higher overall than women	[11,12]
Years lived with disability (YLD)	~26% higher than men despite fewer deaths	-	[11,12]
Sex parity ratios (SPRs) (1990→2021)	Incidence and prevalence parity improved (SPR ~0.61→0.67); mortality and DALY parity declined (SPR ~0.55 in 2021)	-	[11-13]

Sex-Based Pathophysiological Differences in IHD

Women exhibit distinctive pathophysiologic patterns in IHD that notably differ from the classic obstructive CAD paradigm often observed in men [10]. A key contributor is coronary microvascular dysfunction (CMD), a condition in which impaired vasodilation or increased resistance in small coronary vessels causes ischemia despite normal epicardial arteries [14]. CMD is reported far more frequently in women and is frequently overlooked when angiograms fail to show significant stenoses [10]. CMD has been linked to poorer functional outcomes, persistent angina, and reduced response to standard anti-ischemic therapies, underscoring the need to tailor treatment strategies accordingly [10,14].

In parallel, endothelial dysfunction, marked by diminished nitric oxide production, heightened vasoreactivity, and an imbalance of protective endothelial mediators, is more common in women [15]. This dysfunction accelerates both macrovascular and microvascular pathologies and heightens the risk of ischemic events, even in the absence of major arterial blockages [15].

Furthermore, women are more inclined to experience plaque erosion as the precipitating factor for acute coronary syndrome (ACS), rather than plaque rupture [16]. Unlike ruptured plaques, eroded plaques may not produce full vessel occlusion, leading to more subtle or atypical MI presentations [16].

Unique hormonal influences, especially the vasoprotective, anti-inflammatory, and lipid-regulating effects of estrogen, are pivotal. Estrogen’s decline at menopause correlates with an increased prevalence of microvascular disease, shifts in plaque composition, and heightened myocardial vulnerability [17].

As a result, women often display non-classic symptomatology such as fatigue, dyspnea, nausea, indigestion, or escalating anxiety, rather than the stereotypical chest pain. These atypical presentations commonly result in delayed diagnosis and under-treatment [3,18].

Understanding these sex-specific mechanisms is essential for clinical innovation. Incorporating strategies like microvascular function testing, sex-aware imaging protocols, and tailored symptom checklists can enable earlier detection and may drive more equitable management of IHD in women.

Differences in Treatment: PCI and Statin Use

Women are significantly less likely than men to receive evidence-based, guideline-directed treatments such as PCI and statin therapy, even when presenting with comparable clinical indications [1]. 

Women presenting with ACS receive PCI less frequently and later than men, which contributes to worse outcomes [3]. Furthermore, women with STEMI have higher 30-day mortality and major bleeding risk, underscoring differences in pathophysiology and therapeutic responses [19].

Furthermore, fractional flow reserve, a key tool in assessing coronary artery function, is used less often in women than in men [20], indicating a potential gender bias in evaluating coronary physiology. Similarly, women are less likely to be prescribed statin therapy following a STEMI, which can adversely impact their recovery outcomes [21]. One analysis showed that women were 15.8% less likely to receive high-intensity statins after myocardial infarction [18], and up to 10-20% less likely to undergo PCI or coronary angiography following STEMI [4]. This consistent treatment gap highlights a concerning disparity in cardiovascular care, suggesting that factors beyond clinical presentation, such as gender bias, may be influencing medical decision-making.

Cardiac Rehabilitation Disparities

Cardiac rehabilitation (CR) is a cornerstone of secondary prevention in IHD, yet women remain significantly under-referred and under-enrolled in these programs following myocardial infarction [22]. Data show that women are 36% less likely than men to be referred to CR and are 40-50% more likely to drop out or not enroll after referral [23,24]. National registry data consistently demonstrate that women are referred to CR at lower rates than men and, even when referred, face higher rates of non-enrollment and non-completion [22,23]. These disparities persist despite equivalent or greater benefit from CR participation in women.

Several barriers uniquely impact women’s participation in CR, including caregiving responsibilities, limited transportation, older age at first MI, lower socioeconomic status, and greater prevalence of anxiety and depression [22,24]. Additionally, many programs lack scheduling flexibility, childcare support, or psychosocial resources tailored to women's needs. The absence of culturally and gender-sensitive CR models contributes to a perception that the programs are not designed for women, which can discourage engagement [24]. This underrepresentation also limits statistical power in studies assessing CR outcomes for women, affecting the generalizability of findings and potentially skewing sex-specific analyses.

Despite these challenges, evidence shows that women experience significant improvements in functional capacity, symptom burden, and quality of life when they do complete CR, comparable to or greater than their male counterparts [25]. Interventions such as home-based or telehealth CR have been shown to improve participation rates, while women-focused CR programs incorporating mental health, flexibility, and social support yield greater adherence and outcomes [26]. These outcomes underscore the importance of improving both access and adherence.

To bridge these gaps, multi-faceted interventions, including physician endorsement, flexible delivery formats (e.g., home-based or telehealth CR), and women-focused program design, are essential [26]. Programs that explicitly integrate these models have demonstrated up to 25-40% increases in female CR enrollment and completion [26]. Integrating gender equity as a core element of CR delivery may reduce post-MI disparities and improve long-term cardiovascular outcomes in women.

Unique Conditions in Women: MINOCA and SCAD

MINOCA and SCAD are cardiovascular conditions that disproportionately affect women, particularly those under the age of 50. MINOCA accounts for 6-8% of all MIs, but over 70% of these cases occur in women [6,27]. SCAD is a leading cause of MI in women under 50 and is frequently misdiagnosed without intracoronary imaging [5]. SCAD is recognized as a leading cause of MINOCA in this younger female population and often necessitates a high degree of clinical suspicion for accurate diagnosis [5]. The higher incidence of MINOCA among women shows the need for more focused research efforts and the development of sex-specific treatment approaches [6,27]. Due to the distinct pathophysiological mechanisms and presentation in women, managing both MINOCA and SCAD effectively calls for individualized care plans [27]. Addressing these differences is crucial for improving outcomes and reducing disparities in cardiovascular care for women.

Pharmacotherapy and Imaging Considerations

Women’s pharmacologic treatment needs differ due to drug intolerance and adverse reactions, particularly in angina and ischemia with non-obstructive CAD [28]. These differences are not only due to physiological factors but also to how their disease presents clinically. In cases of myocardial infarction, women frequently exhibit unique symptoms and responses to medication, which can impact treatment effectiveness [29]. These atypical presentations may lead to delays in administering evidence-based therapies such as antiplatelet agents and statins, as providers may lack confidence in applying traditional risk algorithms to non-classical symptomatology [29]. These disparities may be influenced by implicit provider bias, differential thresholds for catheterization, and outdated institutional protocols that underemphasize sex-specific presentations [29-31].

Advanced imaging modalities aid in better identification and management of IHD in women, especially in complex cases involving MINOCA and INOCA [7,4].

Summary of Clinical Challenges and Interventions

Table 2 summarizes key diagnostic and therapeutic challenges in women with IHD and outlines sex-specific, evidence-based approaches to address them.

**Table 2:** Clinical Barriers and Sex-Specific Solutions in Ischemic Heart Disease (IHD) PCI: Percutaneous Coronary Intervention

Issue	Challenge in Women	Evidence-based Solution
Symptom presentation	Atypical/non-chest pain symptoms	Use of standardized symptom protocols [30]
Diagnostic underuse	Less referral for imaging and catheterization	Clinical decision tools to ensure referral equity [31]
Treatment disparities	Less PCI and statin use	Sex-aware protocols and continuing education [32]
Trial underrepresentation	~26% female in cardiovascular trials	Inclusion mandates and female leadership [33]

Barriers to Equitable Care

Socioeconomic factors, provider bias, and systemic barriers significantly deepen gender disparities in IHD. Women, especially those from lower socioeconomic backgrounds, show delayed referrals, misdiagnosis, and suboptimal treatment access. It is important to note that sex-based disparities in IHD may be further stratified by race, ethnicity, and socioeconomic status. For instance, Black and Hispanic women face even greater delays in diagnosis and reduced access to care, compounding these risks [34]. For example, a socioeconomic disparity review shows that low socioeconomic status imposes higher IHD risk for women, due to psychosocial and behavioral factors linked to poverty compounded by less access to preventive care [34]. This same study highlights provider-level bias and health system structural inequities as instrumental in this risk gap.

Referral bias is well documented: women are less likely to be referred for cardiac imaging or catheterization than men, even after adjusting for clinical presentation [35]. Similar disparities extend to CR referral and follow-up care, where financial strain, lack of childcare, and work constraints disproportionately affect women. These factors create cumulative disadvantage, a “failure to screen” women for IHD and delay necessary interventions.

Underrepresentation in clinical trials limits the applicability of evidence-based treatments for women. A systematic review of HF trials revealed that women comprised only 26% of participants, far below their population prevalence; trials often under-enrolled and under-screened women [33]. Barriers include restrictive eligibility criteria, logistical challenges, and trial leadership that lacks female representation, while women-led trials included more women [33].

Future Directions and Research Gaps

Efforts to close gender gaps in IHD must prioritize equity in research participation and analysis. Clinical trials continue to under-represent women despite CVD being the leading cause of death for both sexes. A large-scale review of 740 cardiovascular trials conducted between 2010 and 2017 found that women comprised only 38.2% of participants, indicating systemic under-inclusion [36]. This gap is even more pronounced in heart failure trials, where women represent just 28-29% of participants, despite comparable disease prevalence [37,38].

Proposed actions to address gender disparities in IHD research include redesigning eligibility and consent processes to better accommodate women’s needs related to childcare, travel, and employment [39]. Increasing female leadership in clinical trials has also been shown to correlate with higher female enrollment, making it a key strategy for improving representation [40,41]. Moreover, although many trials now include women, there remains a critical gap in sex-disaggregated analysis and reporting, which is essential for understanding sex-specific outcomes [42]. Leveraging initiatives such as the NIH’s Sex as a Biological Variable (SABV) policy and the White House Women’s Health Research Innovation Initiative can help ensure that sex-informed study design becomes a standard in cardiovascular research [43].

Emerging diagnostic criteria, for example MINOCA and SCAD, necessitate prospective validation. Comparative imaging studies, including CMR and PET, show promise in women with non-obstructive IHD but remain underutilized in large cohort trials [10]. These care plans often differ from traditional MI protocols; for example, routine anticoagulation and aggressive PCI may be contraindicated or avoided in SCAD to prevent vessel propagation or perforation [27,44]. Similarly, although SCAD-focused registries have expanded our understanding, most are based on case series; thus, there is an urgent need for dedicated prospective cohorts with long-term follow-up to inform sex-tailored treatment strategies [44].

Discussion

This review consolidates evidence demonstrating persistent gender disparities in the diagnosis and management of IHD. Despite having similar or greater cardiovascular risk, women are consistently underdiagnosed. This underdiagnosis is largely due to atypical symptom presentation, such as fatigue or dyspnea, which do not always align with standard diagnostic criteria. Non-obstructive coronary presentations, including MINOCA and SCAD, further complicate recognition and often go undetected. Additionally, the limited use of advanced imaging techniques in women contributes to missed or delayed diagnoses. The diagnostic gap is further widened by institutional barriers such as the lack of electronic clinical decision support tools, sex-specific diagnostic thresholds, and audit-feedback mechanisms aimed at tracking sex-based disparities. Treatment gaps, such as the underutilization of PCI and statins, continue to contribute to poorer short- and long-term outcomes for women.

Women with STEMI experience a higher risk of complications such as bleeding, underscoring the need for sex-specific therapeutic protocols that balance efficacy and safety [3,18]. This elevated risk may reflect differences in vascular biology, body size, and response to antithrombotic therapies, which are often standardized based on male populations. Additionally, pathologies like MINOCA and SCAD, which are more common in women, further complicate diagnosis and treatment [5,20,26]. These conditions demand heightened clinical suspicion and individualized management to avoid misdiagnosis and ensure appropriate care. Individualized care in SCAD, for example, often avoids anticoagulation or PCI due to risks of vessel propagation-strategies that contrast with traditional MI protocols [27].

To bridge these gaps, healthcare systems must prioritize the integration of sex-specific guidelines into routine practice, particularly in emergency and cardiology settings. Increasing provider education on the distinct presentations and risks faced by women with IHD is essential to reducing diagnostic delays and treatment bias. In addition to education, system-wide interventions such as quality metrics tied to reimbursement, electronic referral alerts, and institutional audits may facilitate adoption of equitable practices. Additionally, professional societies should re-evaluate current cardiovascular guidelines to ensure they reflect emerging evidence on female-specific pathophysiology and treatment responses. For instance, both AHA and ESC guidelines could benefit from greater inclusion of sex-stratified algorithms for MINOCA, CMD, and statin intolerance, as well as female-specific risk calculators. The need for change is also supported by recent studies revealing differences in endothelial function, plaque morphology, and microvascular dysfunction unique to women, which challenge the traditional obstructive CAD paradigm [10,14-16]. Research efforts must also focus on improving the inclusion of women in cardiovascular trials, enabling more robust sex-disaggregated analyses that inform tailored interventions. Trial designs should incorporate sex-based enrollment quotas, pre-specified subgroup analyses, and adjusted sample size calculations to power meaningful sex-stratified outcomes. Policy mechanisms such as NIH SABV compliance monitoring, journal-level mandates for sex-disaggregated reporting, and regulatory agency incentives (e.g., FDA fast-track designation) may accelerate these goals. Likewise, patient education and advocacy initiatives should be amplified to empower women in shared decision-making and increase awareness of atypical IHD symptoms. Without these coordinated efforts in practice, education, policy, and research, gender disparities in cardiovascular outcomes are likely to persist.

This review is limited by its reliance on existing literature, which itself reflects the historical underrepresentation of women in cardiovascular clinical trials. As a result, many treatment recommendations and outcome data are based predominantly on male populations, potentially limiting their generalizability to women. Additionally, while efforts were made to include only high-quality, peer-reviewed sources, variations in study design, population size, and endpoint definitions across the included studies may introduce heterogeneity. No formal systematic review methodology was applied, and selection bias may exist due to preferential citation of more widely available or frequently cited studies. The review also does not include original patient-level data or meta-analytic synthesis, which restricts the ability to draw quantitative conclusions about risk differentials. Finally, many of the cited studies lack sex-disaggregated outcomes, which hinders precise evaluation of gender-specific responses to diagnostic tools and therapies. Future research should prioritize implementation studies that test the effectiveness of tailored interventions such as home-based or psychosocially supported CR programs for women, and mandate sex-specific reporting standards across all phases of cardiovascular research.

## Conclusions

Gender disparities in IHD continue to result in delayed diagnosis, less aggressive treatment, and poorer outcomes for women despite comparable or greater cardiovascular risk profiles. The persistence of these inequities is driven by multiple factors, including atypical symptom presentation, underuse of diagnostic imaging, lower rates of PCI and statin therapy, and the frequent underrecognition of female-predominant conditions like MINOCA and SCAD. These patterns reflect not only gaps in clinical practice but also structural issues in research design, education, and care delivery systems. Addressing these disparities requires a paradigm shift in cardiovascular care that centers sex-specific disease mechanisms and ensures equitable application of evidence-based therapies.

To close this gap, health systems must prioritize the incorporation of sex-informed diagnostic and treatment protocols, improve physician education on female cardiovascular health, and expand access to tailored cardiac rehabilitation programs. Equally important is the need for better representation of women in clinical research and the routine inclusion of sex-disaggregated data in study outcomes. Future efforts should also focus on developing and validating tools specifically designed to detect and treat IHD in women, including those with non-obstructive presentations. Only through these coordinated clinical, educational, and research-based interventions can we ensure that all patients receive accurate diagnoses, appropriate care, and the best possible outcomes in the management of IHD.

## Appendices

List of abbreviations

IHD: Ischemic Heart Disease; MINOCA: Myocardial Infarction with Non-Obstructive Coronary Arteries; SCAD: Spontaneous Coronary Artery Dissection; STEMI: ST-Elevation Myocardial Infarction; PCI; Percutaneous Coronary Intervention; INOCA: Ischemia with Non-Obstructive Coronary Artery Disease; CMD: Coronary Microvascular Dysfunction; CR: Cardiac Rehabilitation; MI: Myocardial Infarction; CAD: Coronary Artery Disease; CMR: Cardiac Magnetic Resonance; PET:- Positron Emission Tomography; SABV: Sex as a Biological Variable; HF: Heart Failure; ACS: Acute Coronary Syndrome
